# Genetic characterization of extraintestinal *Escherichia coli* isolates from chicken, cow and swine

**DOI:** 10.1186/s13568-018-0646-8

**Published:** 2018-07-17

**Authors:** Li Chen, Leyi Wang, Afrah Kamal Yassin, Jilei Zhang, Jiansen Gong, Kezong Qi, Roman R. Ganta, Yuanyuan Zhang, Yi Yang, Xiangan Han, Chengming Wang

**Affiliations:** 1 College of Veterinary Medicine, Yangzhou University, Yangzhou, Jiangsu 225009 China; 2 Department of Veterinary Clinical Medicine and the Veterinary Diagnostic Laboratory, College of Veterinary Medicine, University of Illinois, Urbana, IL 61802 USA; 3 Department of Food Hygiene and Safety, Faculty of Public and Environmental Health, University of Khartoum, Khartoum, 11115 Sudan; 4 Poultry Institute, Chinese Academy of Agricultural Sciences, Yangzhou, Jiangsu 225009 China; 5 Anhui Province Key Laboratory of Veterinary Pathobiology and Disease Control, Anhui Agricultural University, Hefei, 230036 China; 6 Center of Excellence for Vector-Borne Diseases (CEVBD), Department of Diagnostic Medicine/Pathobiology, College of Veterinary Medicine, Kansas State University, Manhattan, KS 66506 USA; 7 Shanghai Veterinary Research Institute, Chinese Academy of Agricultural Sciences, Shanghai, China; 8 College of Veterinary Medicine, Auburn University, Auburn, AL USA

**Keywords:** *Escherichia coli*, Antimicrobial resistance, Whole-genome sequencing, MLST sequence type, VFDB, Phylogenetic analysis

## Abstract

Phenotypic determination of antimicrobial resistance in bacteria is very important for diagnosis and treatment, but sometimes this procedure needs further genetic evaluation. Whole-genome sequencing plays a critical role in deciphering and advancing our understanding of bacterial evolution, transmission, and surveillance of antimicrobial resistance. In this study, whole-genome sequencing was performed on nineteen clinically extraintestinal *Escherichia coli* isolates from chicken, cows and swine and showing different antimicrobial susceptibility. A total of 44 different genes conferring resistance to 11 classes of antimicrobials were detected in 15 of 19 *E. coli* isolates (78.9%), and 22 types of plasmids were detected in 15/19 (78.9%) isolates. In addition, whole-genome sequencing of these 19 isolates identified 111 potential virulence factors, and 53 of these VFDB-annotated genes were carried by all these 19 isolates. Twelve different virulence genes were identified while the most frequent ones were *gad* (glutamate decarboxylase), *iss* (increased serum survival) and *lpfA* (long polar fimbriae). All isolates harbored at least one of the virulence genes. The findings from comparative genomic analyses of the 19 diverse *E. coli* isolates in this study provided insights into molecular basis of the rising multi-drug resistance in *E. coli*.

## Introduction

Antimicrobial resistance (AMR) in bacteria is an important issue related to the health of both human and animals (McDermott et al. 2016; Zawack et al. 2016). It has been reported that AMR bacteria are responsible for about 50,000 deaths in people each year in the USA and Europe, and 700,000 global death (Bottacini et al. 2017) (Zhang et al. 2017). Studies suggested that the agricultural use of antimicrobial agents increases the number of human infections caused by drug-resistant bacteria (O’Neill 2015). Thus, the AMR monitoring system should focus not only on humans, but also animal hosts and the associated environments (Zhang et al. 2017).

*Escherichia coli* is a well-known commensal of the gastrointestinal tract of numerous animals, and also involved in intestinal and extraintestinal pathologies (Tenaillon et al. 2010; Croxen and Finlay 2010). *E. coli* show a clonal population structure with the delineation of at least seven main phylogenetic groups (Desjardins et al. 1995; Clermont et al. 2011). The chromosomal elasticity of the strains helps *E. coli* to adapt to different environments (Touchon et al. 2009).

One of the key issues with *E. coli* is its role in the dissemination and emergence of bacterial antimicrobial resistance. Most of the resistance properties emerge from commensal bacteria in the gastrointestinal tract (Andremont 2003) where the bacteria grow to higher density, allowing horizontal transfers of resistance genes between strains from a single species, and even between species and genera. One of the mechanisms involved in the spread of AMR is the emergence of some specific clones that acquire resistance genes, mostly via mobile genetic elements including plasmids, gene cassettes, transposons, and other integrative genetic elements (Woodford et al. 2011).

Because of its high level of discriminatory power, microbial Whole Genome Sequencing (WGS) strategy plays an important role in the investigation and surveillance of foodborne disease outbreak (Kröger et al. 2012). Better understanding of bacterial evolution, outbreaks, and transmission events revealed with the advent of WGS approach has been shown in a number of recent studies as well as from the surveillance of antimicrobial resistance (Zankari et al. 2012). It has significant advantages compared to other commonly used drug resistant testing approaches. WGS as a diagnostic method to detect bacterial antimicrobial resistance is particularly important where congruence exists between phenotype and genotype, and where phenotypic testing is prohibitively slow for slow-growing bacteria. Epidemiology has benefited greatly from high-throughput WGS in the aspects of identifying and tracking drug-resistant organisms as well as of identifying their genetic diversity (Organization 2014).

The main aim of this study is to investigate the genetic diversity and relationship of *E. coli* clinical strains collected from chicken, cows and swine in China. The *E. coli* clinical strains were further characterized and assigned to the unique profiles of virulence factors and antimicrobial resistance genes.

## Materials and methods

### Bacterial strains

A total of 19 *E. coli* isolates (10 from chicken, 5 from swine, 4 from cows) were selected for WGS analysis in this study. The selection of these isolates was based on their significantly different phenotypes of antimicrobial resistance (Yassin et al. 2017a, b) (Table 1).

**Table 1:** Antibiotic resistance phenotype of the 19 clinical *Escherichia coli* strains

Isolate ID	Animal	Total number of antibiotics	Group	Antibiotics																	
				AMP	AMC	CTX	CRO	CAZ	ATM	ETP	AK	CN	S	TE	C	ENR	NA	CIP	SXT	SF	F
ATCC 25922^a^		0	1																		
E433	Chicken	0																			
E535	Cow	0																			
E80	Chicken	1	2											R							
E533	Cow	1												R							
E175	Swine	2	3										R	R							
E2	Chicken	8	4	R								R	R	R	R		R		R	R	
E30	Chicken	9		R			R					R		R		R	R	R	R	R	
E418	Cow	9		R									R	R	R	R	R	R	R	R	
E565	Swine	9		R									R	R	R	R	R	R	R	R	
E393	Chicken	10		R								R	R	R	R	R	R	R	R	R	
E497	Chicken	11	5	R							R	R	R	R	R	R	R	R	R	R	
E530	Cow	11		R		R	R					R	R	R		R	R	R	R	R	
E34	Chicken	13		R		R	R	R	R			R		R	R	R	R	R	R	R	
E100	Chicken	13		R	R		R		R			R	R	R	R	R	R	R	R	R	
E122	Chicken	12		R		R	R					R	R	R	R	R	R	R	R	R	
E205	Swine	13		R		R	R				R	R	R	R	R	R	R	R	R	R	
E543	Chicken	13		R		R	R	R	R				R	R	R	R	R	R	R	R	
E461	Swine	15		R	R	R	R	R			R	R	R	R	R	R	R	R	R	R	
E386	Swine	16		R	R	R	R	R			R	R	R	R	R	R	R	R	R	R	R

*AMP* ampicillin, *AMC* amoxicillin/clavulanic acid, *CTX* cefotaxime, *CRO* ceftriaxone, *CAZ* ceftazidime, *ATM* aztreonam, *ETP* ertapenem, *AK* amikacin, *CN* gentamicin, *S* streptomycin, *TE* tetracycline, *C* chloramphenicol, *ENR* enrofloxacin, *NA* nalidixic acid, *CIP* ciprofloxacin, *SXT* trimethoprim/sulfamethoxazole, *SF* sulfafurazole, *F* nitrofurantoin

^a^*E. coli* (ATCC 25922) served as quality control strain

### Whole genome sequencing and data analysis

Genomic DNA from all the 19 *E. coli* isolates were extracted, end repaired, ligated to specific adaptors and subject to paired-end sequencing using Illumina HiSeq 2500 by PE125 strategy at Beijing Novogene Bioinformatics Technology Company (Beijing, China). After filtering raw reads, the clean reads were de novo assembled into contigs using the CLC Genomics Workbench. The assembled genomes were analyzed by using online software tools provided by the Centre of Genomic Epidemiology (https://www.genomicepidemiology.org/). In addition, CGE ResFinder 2.1 was used to identify the antimicrobial resistance genes in the assembled genomes using (Zankari et al. 2012). The minimum percentage of the gene length detected and the identity threshold was set to be a 90.0% identity for a positive match between a target genome and the reference database.

The MLST server database v1.7 (Larsen et al. 2012) and the Virulence Finder server database v1.2 (Joensen et al. 2014) in the CGE website were used to identify virulence genes and housekeeping genes (*adk*, *fumC*, *icd*, *gyrB*, *mdh*, *purA*, *recA*). The scaffolds of each isolate were incorporated into these tools as described in CGE, with an identity threshold set to be 98%. The profile of replicons of bacterial plasmids was identified by the use of PlasmidFinder-1.3 (Carattoli et al. 2014).

Double index alignment of next-generation sequencing data (DIAMOND) (Buchfink et al. 2015) was applied to align the amino acid sequences against the VFDB database (Chen et al. 2015). The annotation of predicted gene with the description of the best fit was defined as amino acid sequences with alignment length > 90% of its own length and over 20% match identity.

Phylogenomic relationships among strains were assessed based on nucleotide alignments of the core genome gene content, including only the single-copy orthologues. An additional filter for paralogues was applied to the core genome in order to exclude families represented by more than a single member since they do not represent robust evolutionary markers (Gutiérrez and Maere 2014). Gene alignments were conducted using MUSCLE v.3.8.31 (Edgar 2004), followed by construction of a phylogenetic tree for each single-copy gene using the maximum-likelihood (≤5 samples) in PhyML v3.0 (Guindon and Gascuel 2003) and tree concatenation (Bottacini et al. 2017).

### GenBank accession numbers

This Whole Genome Shotgun project 19 *E. coli* isolates investigated in this study has been deposited at DDBJ/ENA/GenBank under the following accession numbers: *E. coli* E565, QETO00000000; *E. coli* E535, QETQ00000000; *E. coli* E533, QETR00000000; *E. coli* E530, QETS00000000; *E. coli* E497, QETT00000000; *E. coli* E461, QETU00000000; *E. coli* E433, QETV00000000; *E. coli* E418, QETW00000000; *E. coli* E393, QETX00000000; *E. coli* E386, QETY00000000; *E. coli* E205, QETZ00000000; *E. coli* E175, QEUA00000000; *E. coli* E122, QEUB00000000; *E. coli* E100, QEUC00000000; *E. coli* E80, QEUD00000000; *E. coli* E34, QEUE00000000; *E. coli* E30, QEUF00000000; and *E. coli* E2, QEUG00000000.

Strain E497 from chicken, one of the multidrug-resistant *E. coli* strains investigated in this study, was deposited at China General Microbiological Culture Collection Center (CGMCC) with a deposit number of CGMCC-10601.

## Results

### Phenotypic analysis of antimicrobial resistance of 19 clinical *E. coli* strains

All 19 clinical *E. coli* isolates, except for E433, E535, E80 and E533, displayed MDR phenotype with resistance to 2–16 antimicrobials (Table 1). In total, we identified 14 resistance patterns, and none of the strains was resistant to ertapenem. Based on antibiogram results, the 19 clinical *E. coli* isolates were divided into five groups (Table 1).

### Genotypic analysis of antibiotic resistance genes

**Fig. 1:**
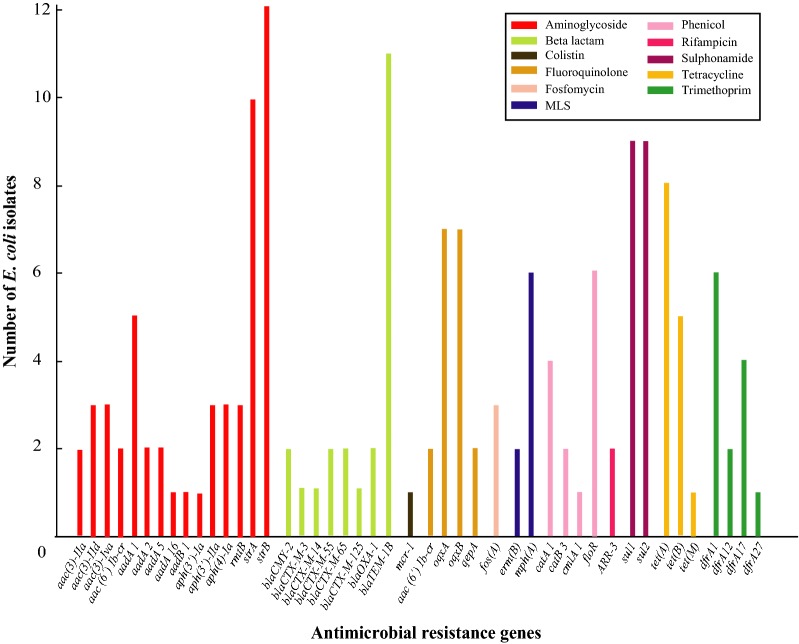
Presence of antibiotic resistance gene in clinical WGS *E. coli* isolates. The X axis shows the antibiotic resistance gene, and the Y-axis is the number of resistance genes in clinical *E. coli i*solates. The colors of the bars denotes resistance to different classes of antimicrobials

Four of 19 strains did not harbor any of the antimicrobial resistance genes analyzed, while the remaining 15 isolates had more than one resistance gene. A total of 44 different antimicrobial resistance genes were identified in these 15 isolates conferring resistance to 11 classes of antimicrobials (Fig. 1). One isolate (5.3%) possess *mcr*-1 gene in a full-length copy of a colistin resistance gene that showed 100% nucleotide similarity to the reference database sequence (Table 2).

**Table 2:** Distribution of antibiotic resistant genes in 19 clinical *E. coli* strains

Antimicrobial	AR gene	E433	E497	E535	E565	E80	E533	E175	E2	E386	E461	E543	E393	E418	E530	E30	E205	E100	E34	E122
Aminoglycoside	*aac(3)*-*IIa*									+			+							
	*aac(3)*-*IId*														+		+	+		
	*aac(3)*-*IVa*																	+	+	+
	*aac(6’)Ib*-*cr*																		+	+
	*aadA1*								+		+	+	+			+				
	*aadA16*																	+		
	*aadA2*															+				+
	*aadA5*									+					+					
	*aadB*										+									
	*aph(3′)*-*Ia*									+										
	*aph(3′)*-*IIa*										+					+		+		
	*aph(4)*-*Ia*																	+	+	+
	*rmtB*															+	+		+	
	*strA*							+	+	+	+	+		+		+	+		+	+
	*strB*							+	+	+	+	+		+	+	+	+	+	+	+
Beta-lactam	*blaCMY*-*2*									+	+									
	*blaCTX*-*M*-*3*														+					
	*blaCTX*-*M*-*14*																+			
	*blaCTX*-*M*-*55*											+							+	
	*blaCTX*-*M*-*65*																	+		+
	*blaCTX*-*M*-*125*															+				
	*blaOXA*-*1*																		+	+
	*blaTEM*-*1B*								+	+	+	+		+	+	+	+	+	+	+
Colistin	*mcr*-*1*																			+
Fluoroquinolone	*aac(6′)Ib*-*cr*																		+	+
	*oqxA*							+		+			+	+		+			+	+
	*oqxB*							+		+			+	+		+			+	+
	*qepA*																+			+
Fosfomycin	*fosA*														+	+		+		
Macrolide, Lincosamide, Streptogramin B	*mph(A)*									+			+		+	+		+	+	
	*erm(B)*																+	+		
Phenicol	*catA1*									+		+		+			+			
	*catB3*																		+	+
	*cmlA1*										+									
	*floR*										+		+			+		+	+	+
Rifampicin	*ARR*-*3*																	+	+	+
Sulphonamide	*sul1*								+		+	+	+		+	+		+	+	+
	*sul2*								+		+	+	+	+		+	+		+	+
Tetracycline	*tet(A)*							+	+		+	+	+		+			+		+
	*tet(B)*					+	+							+			+		+	
	*tet(M)*																+			
Trimethoprim	*dfrA1*								+		+	+	+			+			+	
	*dfrA12*															+				+
	*dfrA17*									+				+	+		+			
	*dfrA27*																	+		
Number of predicted resistance antimicrobial class		0	0	0	0	1	1	3	5	6	6	6	7	7	7	8	8	9	9	9

### Prevalence of plasmid replicons

Through WGS analysis, 22 plasmid replicons were identified in 15 of the 19 isolates. Fourteen isolates harbored multiple plasmid replicons. Eighteen types of *Inc* with different frequencies were found, including *IncA/C2, IncFIA, IncFIB (AP001918), IncFIB(K), IncFIB(pLF82), IncFIC(FII), IncFII, IncFII(pCoo), IncFII(pHN7A8), IncHI2, IncHI2A, IncI1, IncI2, IncN,, IncQ1, IncR, IncX1 and IncY* (Table 3).

**Table 3:** Analysis of plasmids in *E. coli* strains

Plasmid	E80	E433	E497	E535	E530	E533	E565	E34	E122	E175	E386	E393	E418	E30	E2	E461	E205	E543	E100
Col156								+				+			+			+	
Col(BS512)																	+		
Col(MG828)																			+
IncA/C2																+			
IncFIA																	+		
IncFIB(AP001918)							+			+	+		+	+	+	+	+	+	
IncFIB(K)									+	+		+							
IncFIB(pLF82)													+						
IncFIC(FII)							+						+		+	+		+	
IncFII					+			+			+					+	+	+	
IncFII(pCoo)														+					
IncFII(pHN7A8)										+									
IncHI2																			+
IncHI2A																			+
IncI1								+		+		+			+	+	+		+
IncI2									+										
IncN									+		+			+				+	+
IncQ1											+				+		+	+	+
IncR						+								+					
IncX1														+					+
IncY						+							+				+		+
p0111									+			+			+	+		+	
Total number of genotypic plasmids	0	0	0	0	1	2	2	3	4	4	4	4	4	5	6	6	7	7	8

### Virulence genes

At least one virulence gene was detected in all 19 *E. coli* isolates evaluated. Twelve different virulence genes were identified while the most frequent ones were *gad* (glutamate decarboxylase), *iss* (increased serum survival) and *lpfA* (long polar fimbriae) which were identified in 14, 11 and 9 isolates, respectively (Table 4).

**Table 4:** Analysis of virulence genes in different *E. coli* strains

Virulence gene	E2	E30	E34	E80	E100	E122	E175	E205	E386	E393	E418	E433	E461	E497	E530	E533	E535	E543	E565
*astA*		+					+												
*capU*							+												
*celb*	+																		
*cma*									+									+	
*gad*	+	+		+	+		+	+		+			+	+	+	+	+	+	+
*ireA*			+															+	
*iroN*	+								+		+		+					+	+
*iss*	+	+				+		+	+		+		+		+	+		+	+
*lpfA*	+			+	+				+		+	+	+	+					+
*mchF*	+										+		+						+
*stb*							+												
*tsh*	+												+					+	+
Total number of virulence genes	7	3	1	2	2	1	4	2	4	1	4	1	5	2	2	2	1	6	6

### Mlst

The multilocus sequence typing of the isolates is shown in Table 5. Three isolates (E100, E386 and E433) belong to Sequencing Type (ST) 155 while two isolates (E461 and E565) belong to ST 23 and another two isolates (E80 and E533) belong to ST 297. The remaining 12 isolates belong to individual MLST types, ST2505, ST746, ST656, ST10, ST3345, ST4012, ST6856, ST602, ST2111, ST5019, ST548 and ST4753.

**Table 5:** MLST profiles of the *E. coli* isolates

Sample	ST	Allele						
		*adk*	*fumC*	*gyrB*	*icd*	*mdh*	*purA*	*recA*
E2	2505	6	41	12	1	20	13	7
E30	746	10	7	4	8	12	8	2
E34	656	10	7	4	8	8	8	2
E80	297	6	65	32	26	9	8	2
E100	155	6	4	14	16	24	8	14
E122	10	10	11	4	8	8	8	2
E175	3345	64	7	1	369	8	8	6
E205	4012	8	517	1	8	8	8	6
E386	155	6	4	14	16	24	8	14
E393	6856	6	11	4	732	8	8	2
E418	602	6	19	33	26	11	8	6
E433	155	6	4	14	16	24	8	14
E461	23	6	4	12	1	20	13	7
E497	2111	6	4	32	88	7	7	6
E530	5019	6	518	4	10	7	8	6
E533	297	6	65	32	26	9	8	2
E535	548	10	11	4	8	81	8	2
E543	4753	6	612	5	28	1	1	2
E565	23	6	4	12	1	20	13	7

### VFDB analysis

In this study, WGS of these 19 isolates and the analysis identified 111 potential virulence factors. Fifty-three of these VFDB-annotated genes were carried by all these 19 isolates. The VFDB-annotated genes are responsible to adherence, autotransporter, invasion, iron uptake, toxins, secretion system and secretion system related effectors. Different potential virulence factors with different abundance were observed, and the most abundant virulence factors was associated with adherence. In addition, some of the isolates contained pathogenic *E. coli* virulence factors, such as *Chu* (isolate E80) present in *Enterohemorrhagic E. coli* (EHEC) and *Per* (isolates E30, E205, E433, E533) present in *Enteropathogenic E. coli* (EPEC).

### Phylogenomic analysis

**Fig. 2:**
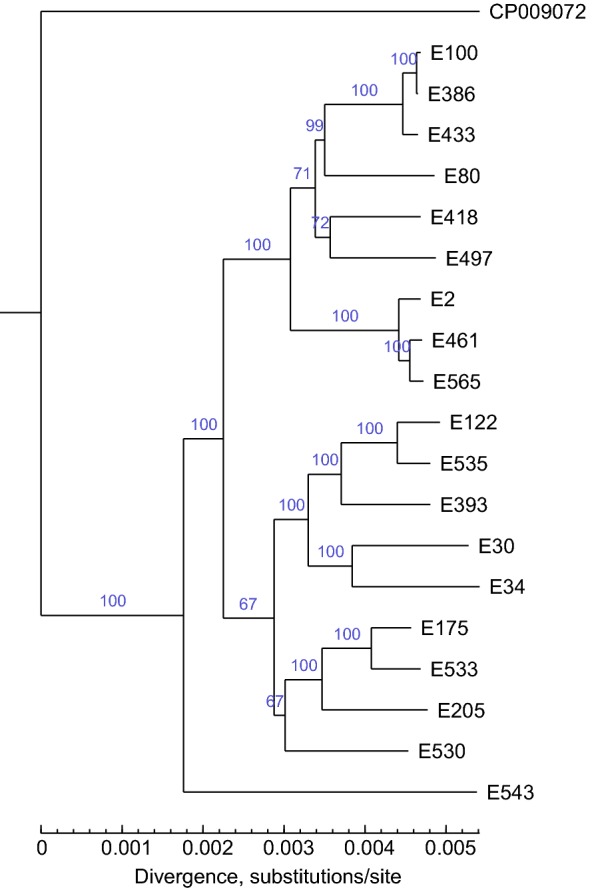
Phylogenomic analysis of *E. coli* isolates. Phylogenetic Supertree computed on concatenated single-copy core genes and using the Neighbor-Joining method showing the existing relationship between 19 *E. coli* species. CP009072 was used as the outgroup. Evolutionary analyses were conducted in TreeBest. The numbers on the branch indicate branch credibility. The branch length shows the size of the evolutionary distance which calculates in the units of the number of base substitutions per site

In this study, a total of 2858 core genes are present as single copies in isolates, and the resulting phylogenetic tree was computed using the 19 clinical *E. coli* isolates sequenced while *E. coli* CP009072 serves as an outgroup (Fig. 2). The phylogenetic analysis provided complete resolution of relationships among all isolates sampled, with maximum support (100) for all nodes. The *E. coli* isolates are clustered in seven phylogenetic groups, and E543 was derived firstly from the lineage of the remaining members of the subfamily (Fig. 2). The isolates of E100, E386, E433 and E80 appear to be monophyletic and more closely related to E418 and E497 than to E2, E461 and E565. *E. coli* comprised this clade plus another in which E122, E535 plus E393 and E30 plus E34 (both monophyletic) were together adjacent to E175, E533, E205 and E530 (Fig. 2).

## Discussion

The genetic characteristics of clinical *E. coli* isolates can provide useful information about the potential for causing disease and resistance to treatment. In this study, we analyzed the WGS of *E. coli* isolates obtained from chickens, swine, and cows in Jiangsu province, China. These isolates exhibited a diverse range of genetic profiles.

These isolates have quite comprehensive profiles being resistant to beta-lactams, quinolones, aminoglycosides and colistin. Most of the strains displayed consistent antibiogram and antibiotic resistance gene profiles, but some of them showed inconsistency between the phenotype and the genotype. For instance, there is not any antibiotic resistance gene cluster profile displayed in the isolates E497 and E565 which however displayed resistance to a panel of antibiotics (Tables 1, 2). This is likely due to an unidentified regulatory mechanism in these isolates. In this study, one of the most important antimicrobial resistance genes identified was *mcr*-*1*, mediating resistance against colistin (Liu et al. 2016; Yassin et al. 2017a, 2017b). The plasmid-borne colistin resistance gene *mcr*-*1* was found in bacterial strains of both humans and animals (Liu et al. 2016). This is of a great public health concern as these colistin is considered as “last-resort” drugs for human infections caused by multi-drug resistant *Enterobacteriaceae* (Shaheen et al. 2013). The identified *mcr*-*1* gene by WGS in this study was verified by PCR and the *mcr*-1-positive *E. coli* was verified to be resistant to colistin (Yassin et al. 2017a, 2017b).

Plasmids as diverse and self-replicating extrachromosomal elements encode a variety of traits which include antimicrobial resistance, virulence, and environmental adaptability. Plasmids also plays a major role in bacterial adaptation to environmental (Smets and Barkay 2005). *Inc* plasmids target the replicons of the major plasmid families occurring in *Enterobacteriaceae* (HI2, HI1, I1-ã, X, L/M, N, FIA, FIB, FIC, W, Y, P, A/C, T, K, B/O) (Carattoli 2009). Currently, there are 27 known *Inc* groups occurring among the *Enterobacteriaceae* family (Frost et al. 2005; Carattoli 2009). Classification of plasmids into Inc groups is desirable because specific plasmid types have been associated with virulence and/or antimicrobial resistance (Gilmour et al., 2004; Hopkins et al. 2006; Carattoli et al. 2014). In this study, 18 types of *Inc* plasmids were detected. *IncI 1* plasmid has been shown to contribute to adhesion and invasion of shiga-toxigenic *E. coli* due to presence of a cluster encoding IV pili (Kim and Komano 1997). While plasmids mediating antimicrobial resistance in *Enterobacteriaceae* is highly variable, some plasmid families are largely prevalent and also prevalently associated with specific resistance genes (Carattoli 2009).

As shown in Table 5, 19 clinical *E. coli* isolates possess 15 different ST types based on the MLST analysis. ST10 is one of the important multilocus sequence types possessed by one isolate (E122) in our findings, which confers resistant to colistin and is often reported as antibiotics against ESBL-producing *E. coli* (Chen et al. 2016). This ST type is also commonly found in chickens, other animals and humans (Chen et al. 2016) and this is consistent with our findings that this strain was isolated from chicken.

Among 12 different virulence genes possessed by all isolates, the highest frequencies appeared was *gad*, *iss* and *lpfA* which were frequently reported in pathogenic *E. coli* isolates (Bergholz et al. 2007; Solà-Ginés et al. 2015; Malik et al. 2017). Thirteen isolates which carried these gene harbored multiple antimicrobial resistance genes, and 9 of them carried genes for *lpfA* virulence and that has been described to be a potential virulence marker for pathogenic *E. coli* (Petty et al. 2014). However, the presence of a single or multiple virulence genes in an *E. coli* strain does not warrant that a strain is pathogenic unless that strain has the appropriate combination of the virulence genes to cause infections in the hosts (Boerlin et al. 1999). Pathogenic *E. coli* uses a complex multi-step mechanism of pathogenesis involving a number of virulence factors which consists of attachment, host cell surface modification, invasion, a variety of toxins and secretion systems, eventually leading to death of the target host cells (Kaper et al. 2004). Thus, virulence genes are ideal targets for determining the pathogenic potential of a given *E. coli* isolate (Kuhnert et al. 2000).

Virulence factors are important for microbial pathogenesis. A mutation of a virulence factor from a virulent pathogen will attenuate the pathogen strain (Volk et al. 1995). However, virulence factors may also exist in attenuated and even avirulent strains (Chen et al. 2015).Our study attempted to characterize the clinical isolates for the presence of virulence associated genes by comparison against a database collection of virulence factors, VFDB. In our study, we observed that 53 of the VFDB-annotated genes were shared within 19 clinical *E. coli* isolates. The most abundant adherence found in our isolates maybe related to the *IncI 1 plasmids*, which can encode the type IV pili. These virulence factors, along with their epidemic ability and resistance determinants, may have favored the dissemination of plasmids belonging to *IncI 1* plasmid family (Carattoli 2009).

Based on nucleotide alignments of the core genome of individual strains, phylogenomic investigation allowed us to deduce the evolutionary relationships between strains while 16S rRNA sequence-based phylogeny does not provide sufficient resolution at the intra-species level (Ventura et al. 2006). The phylogenetic analysis of the 19 clinical *E. coli* isolates in our study showed no group correlations between the isolates from the same species or numbers of antibiotic resistance genes possessed by them. MLST analysis showed that strains E100, E386, E433 and strains E461, E565 belonged to ST155 and ST23, respectively, which are phylogenetically similar. Unlike comparative genomics-derived clustering which is based on the presence-absence of genes, phylogenomic analysis is based on sequence alignment of core genes and for this reason it is more suitable for an in depth investigation of phylogenetic relationships between closely related taxa (Bottacini et al. 2017).

This study confirmed that multiple drug resistance of most of the clinical *E. coli* isolates were probably due to the presence of different plasmids. Virulence genes carried by these isolates can increase potential risks on the health of human and animals. Virulence factors associated with adherence have the most abundance in Virulence Factors of Pathogenic Bacteria analysis. With the rapidly falling cost and turnaround time as well as availability of more user-friendly software, WGS promises to be transformative for rapid surveillance and genotypic antimicrobial susceptibility testing for microbes that are difficult to grow, and has great benefits in combination with phenotypic methods. The findings from comparative genomic analyses of the 19 diverse *E. coli* isolates provided insights into molecular basis of the rising multi-drug resistance in *E. coli*.

The WGS-based characterization of multidrug-resistant *E. coli* from extraintestinal infections in three animal species in this study revealed a diverse range of *E. coli* STs, and demonstrated the emergence and persistence of particular multidrug-resistant strains, which may have a competitive advantage in fitness under antimicrobial selection pressure compared with previous strains. Surveillance of the emergence and spread of dominant multidrug-resistant isolates with unique plasmids, resistance genes, virulence genes and STs may assist veterinarians in developing improved strategies for treatment and prevention of infections for which the choice of antimicrobials is limited.

## Abbreviations

WGS: whole-genome sequencing
AMR: antimicrobial resistance
MLST: multi locus sequence typing
CGE: Centre of Genomic Epidemiology
VFDB: virulence factor database
DIAMOND: double index alignment of next-generation sequencing data
EHEC: *Enterohemorrhagic E. coli*
EPEC: *Enteropathogenic E. coli*
HGT: horizontal gene transfer
ESBL: extended-spectrum β-lactamase
*mcr*: mobile colistin resistance
*astA*: arginine succinyltransferase A
*capU*: Cappuccino, hexosyltransferase homolog
*celb*: Caldocellum saccharolyticum
*cma*: colicin-M
*gad*: glutamate decarboxylase alpha
*ireA*: iron-responsive element A
*iss*: increased serum survival
*lpfA*: long polar fimbriae A
*mchF*: microcin transporter protein F
*stb*: heat-stable enterotoxin B
*tsh*: temperature-sensitive hemagglutinin
